# Measuring by Hand: Arsenic Picked Up from the Playground

**Published:** 2004-10

**Authors:** Scott Fields

Several nations today ban or severely restrict the use of wood preserved with chromated copper arsenate (CCA), but many existing structures still remain—for example, about 70% of existing U.S. single-family homes and 14% of public playgrounds incorporate CCA-treated wood. In recent years, scientists have studied how arsenic leaches from CCA-treated wood, but they have only inferred exposure levels from measurements of arsenic concentrations in soil and sand near treated wood structures. In this issue, Elena Kwon of the University of Alberta and colleagues report on direct measurements they made of arsenic on the hands of children playing in playgrounds, some with CCA-treated wood structures and others without **[*EHP* 112:1375–1380]**. The team reports that although playing on treated structures increases the amount of arsenic on children’s hands, washing the children’s hands after playing may be enough to avoid the health risks associated with CCA.

For several decades, CCA-treated wood was widely used in the United States, Canada, and other countries for playground equipment, fences, and backyard decks. Bans and restrictions on the use of CCA-treated wood have been driven largely by concerns that treated wood could release chromium and arsenic, posing risks to human health. Especially vexing was the possibility that children who contacted CCA-treated wood structures were, because of their propensity for hand-to-mouth contact, especially at risk for ingesting arsenic. Although touching treated wood will not liberate the 70- to 170-milligram dose of arsenic that is fatal to humans, ingesting lower doses of the substance has been linked to several cancers and other ailments.

The scientists measured arsenic on the hands of 130 children who visited 16 public playgrounds in Edmonton, Canada, over the period 5–21 August 2003. They tested all children who visited the playgrounds during randomized observation times and whose parents allowed them to participate in the study. The children averaged 4.75 years of age and spent an average of 1.25 hours on the playground.

When each child was finished playing, his or her hands were rinsed for 1 minute in a Ziploc bag of deionized water. The water and any soil/sand rinsed from the child’s hands were analyzed separately in the laboratory for arsenic content. The team also collected soil/sand samples from each playground; samples from near the structures provided a measure of the arsenic that had leached from the wood, while those taken far from the structures indicated how much arsenic was present naturally.

In comparing playgrounds with and without CCA-treated wood structures, the team found no statistically significant difference in the amount of arsenic in the soil/sand samples or in the soil/sand washed from the children’s hands. However, children who had played in the treated-wood environment had an average of 0.50 micrograms of soluble arsenic rinsed from their hands—more than five times as much as the children who did not play on treated structures.

EPA research indicates that ingestion, rather than inhalation or dermal absorption, is the primary route of exposure related to arsenic-related ailments. Children aged 2–6 typically ingest about half of whatever they collect on their hands. But even assuming that the children in the study managed to ingest all of the arsenic on their hands, their average dosage was less than the average Canadian child’s daily dose of arsenic through food and water (about 0.6 micrograms per kilogram body weight).

The scientists also found that the first rinsing removed most of the arsenic from the children’s hands. That could be the prescription for parents whose children frequent playgrounds with treated-wood structures—and who want to play it safe.

## Figures and Tables

**Figure:**
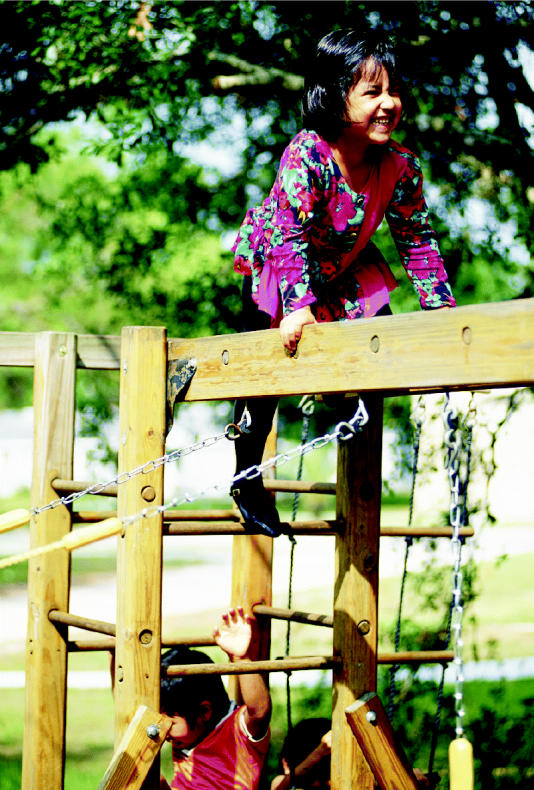
**Picked up on the playground.** Arsenic from treated wood play structures is transferred to children’s hands, but washing can remove most of it.

